# Effectiveness of a 23-valent pneumococcal polysaccharide vaccine for the prevention of pneumococcal pneumonia in the elderly with chronic respiratory diseases: a case–control study of a single center

**DOI:** 10.1186/s12890-021-01491-w

**Published:** 2021-04-16

**Authors:** Toshihiro Masuda, Eiji Nakatani, Toshihiro Shirai, Taisuke Akamatsu, Kanami Tamura, Shingo Takahashi, Yuko Tanaka, Hirofumi Watanabe, Yoshinari Endo, Takahito Suzuki, Mika Saigusa, Akito Yamamoto, Satoru Morita, Yoko Sato, Kazuhiro Asada

**Affiliations:** 1 Department of Respiratory Medicine, Shizuoka General Hospital, 4-27-1, Shizuoka, Japan; 2 Division of Clinical Biostatistics, Research Support Center, Shizuoka General Hospital, 4-27-1, Kita-ando, Aoi, Shizuoka, 420-8527 Japan; 3 Graduate School of Public Health, Shizuoka Graduate University of Public Health, 4-27-2 Kitaando, Aoi-ku, Shizuoka, 420-0881 Japan

**Keywords:** Chronic respiratory disease, Elderly, Pneumococcal polysaccharide vaccine, Pneumococcus, Pneumonia, Vaccine

## Abstract

**Background:**

The effectiveness of the 23-valent pneumococcal polysaccharide vaccine (PPSV23) in preventing pneumococcal pneumonia has been controversial.

**Methods:**

To evaluate the effectiveness of the PPSV23 in elderly outpatients with chronic respiratory diseases, we carried out a case–control study, including 4128 outpatients aged ≥ 65 years, in the respiratory department.

**Results:**

There were 320 vaccinated patients, of which 164 were diagnosed with pneumococcal pneumonia. The adjusted odds ratio was 0.39 (95% confidence interval (CI), 0.17 to 0.89). In the subsets consisting of age groups ≥ 70 and ≥ 75 years, the adjusted odds ratio (95% CI) was respectively 0.16 (0.04 to 0.67) and 0.15 (0.02 to 1.12).

**Conclusion:**

This real-world study suggests that PPSV23 can be useful in preventing pneumococcal pneumonia in the elderly with chronic respiratory diseases.

**Supplementary Information:**

The online version contains supplementary material available at 10.1186/s12890-021-01491-w.

## Background

*Streptococcus pneumoniae* can cause pneumonia and invasive pneumococcal diseases (IPDs), which result in considerable morbidity and mortality worldwide [1, 2]. The Advisory Committee on Immunization Practices recommends the use of the 23-valent pneumococcal polysaccharide vaccine (PPSV23) or the 13-valent pneumococcal conjugate vaccine (PCV13) for all the elderly (age ≥ 65 years) and for immunocompromised adults [3]. In Japan, the PPSV23 coverage of people aged ≥ 65 years was 32.4% in 2019 [4], and that of the PCV13, which is not covered by Japanese universal health insurance, has not been reported because of the extremely small number of people vaccinated. The effectiveness of the PPSV23 in preventing IPD has been reported, its effectiveness in preventing pneumococcal pneumoniae has been inconsistent [5–7]. Some researchers have targeted both healthy individuals and patients with various diseases at nursing home residences [6, 8].

We hypothesized that the PPSV23 would be useful in preventing pneumococcal pneumonia in elderly outpatients with chronic respiratory diseases. This study aimed to assess the effectiveness of the PPSV23 among elderly outpatients in clinical practice.

## Methods

### Study design and population

This study was a retrospective case–control design. The target population was defined as outpatients aged ≥ 65 years, with chronic respiratory diseases, treated between 2015 and 2017 in the respiratory department of Shizuoka General Hospital. From this sample, the case and control groups consisted of patients with and without pneumococcal pneumonia, respectively. Patients who had been vaccinated with PCV13 were excluded.

### Diagnosis of pneumococcal pneumonia

Respiratory physicians diagnosed pneumonia based on clinical findings such as fever, hypothermia, chills, cough, sputum production, pleuritic chest pain, fatigue, tachypnea, white blood cell count > 9300, or < 4000 cells/mm^3^, and new pulmonary infiltrates on chest radiography [2]. In this study, all patients with pneumonia met these criteria. Pneumococcal pneumonia was diagnosed based on the positive results of urine pneumococcal antigen and sputum culture, but a negative blood culture for pneumococcus.

### Definitions

The chronic respiratory diseases in this study included lung cancer, asthma, chronic obstructive pulmonary disease (COPD), interstitial pneumonia, pulmonary non-tuberculous mycobacteriosis (NTM), pulmonary tuberculosis, and others. The history of PPSV23 vaccination was obtained from medical records and declarations by patients or their families. Patients were considered vaccinated when they had received the PPSV23 within five years prior to the diagnosis of pneumonia. Patients without medical records or whose families had no knowledge of their vaccination statuses were considered unvaccinated.

### Statistical analysis

The chi-squared tests for categorical variables and t-tests for continuous variables were used in comparing both groups. To evaluate the effectiveness of the PPSV23, we performed a logistic regression analysis, and then the odds ratio (OR), 95% confidence interval (CI), and *p* value (based on Wald test) were calculated. The adjusted OR was estimated by the quantile stratification method of propensity scores. The propensity score was estimated using multivariate logistic regression models with potential confounders as covariates, which included all variables of Table 1. We also made two subsets: those ≥ 70 years, and ≥ 75 years, and compared their adjusted ORs with that in all patients. As a sensitivity analysis, we estimated double-robust adjusted OR in case–control studies under causal inference [9], and we confirmed whether the point estimation of OR, as mentioned above, was overestimating the effect.

**Table 1:** Characteristics of patients with and without pneumococcal pneumonia

Variable and category (reference)	Case group (n = 164)	Control group (n = 4,054)	*p* value
Age, years^a^	76.2 ± 7.3	75.1 ± 6.7	0.127
65–69	32 (19.5)	984 (24.3)	0.153
70–74	44 (26.8)	1,098 (27.1)	
75–79	36 (22.0)	950 (23.4)	
80 +	56 (34.1)	1,063 (26.2)	
Male (vs. female)	113 (68.9)	2,525 (62.3)	0.100
Smoking			0.001
Non-smokers	37 (22.6)	1,198 (29.6)	
Current smokers	108 (65.9)	2,068 (51.0)	
Ex-smokers	19 (11.6)	788 (19.4)	
Chronic respiratory diseases (vs. absent)			
Asthma	30 (18.3)	685 (16.9)	0.671
COPD	42 (25.6)	959 (23.6)	0.574
Lung cancer	45 (27.4)	1,594 (39.3)	0.002
Interstitial pneumonia	29 (17.7)	703 (17.3)	0.916
NTM	10 (6.1)	449 (11.1)	0.054
Others^b^	48 (29.3)	746 (18.4)	0.001
Diabetes (vs. absent)	65 (39.6)	1,209 (29.8)	0.023
Chronic heart disease (vs. absent)	95 (57.9)	1,891 (46.6)	0.005
Chronic kidney disease (vs. absent)	13 (7.9)	299 (7.4)	0.761
Systemic corticosteroid user (vs. absent)	65 (39.6)	1,189 (29.3)	0.007

Values are expressed as numbers and proportions in parentheses

^a^Mean ± SD

^b^Other chronic respiratory diseases included chronic pulmonary aspergillosis, old pulmonary tuberculosis, sarcoidosis, and chronic cough

COPD, chronic obstructive pulmonary disease; NTM, non-tuberculous mycobacteriosis

To confirm the efficacy of the vaccine for each age group (65 to < 70 years, 70 to < 75 years, and ≥ 75 years), crude ORs, ORs adjusted for risk factors, and their 95% confidence intervals were calculated. Risk factors for pneumococcal pneumonia were identified as follows. Variables for which the *p* value of the comparison test between the case and control groups was less than 0.05 were considered as candidate risk factors, and these variables were entered into a multivariate logistic regression model. In this multivariate model, variables with *p* values less than 0.05 were identified as risk factors. Furthermore, to extract variables containing different categories of pneumococcal pneumonia proportions in different age groups, an interaction term test using a logistic regression model was performed.

A *p* value of < 0.05 was considered to be statistically significant. Statistical analyses were performed using SAS version 9.4 (SAS Institute, Cary, NC, USA).

## Results

### Patients background

**Fig. 1:**
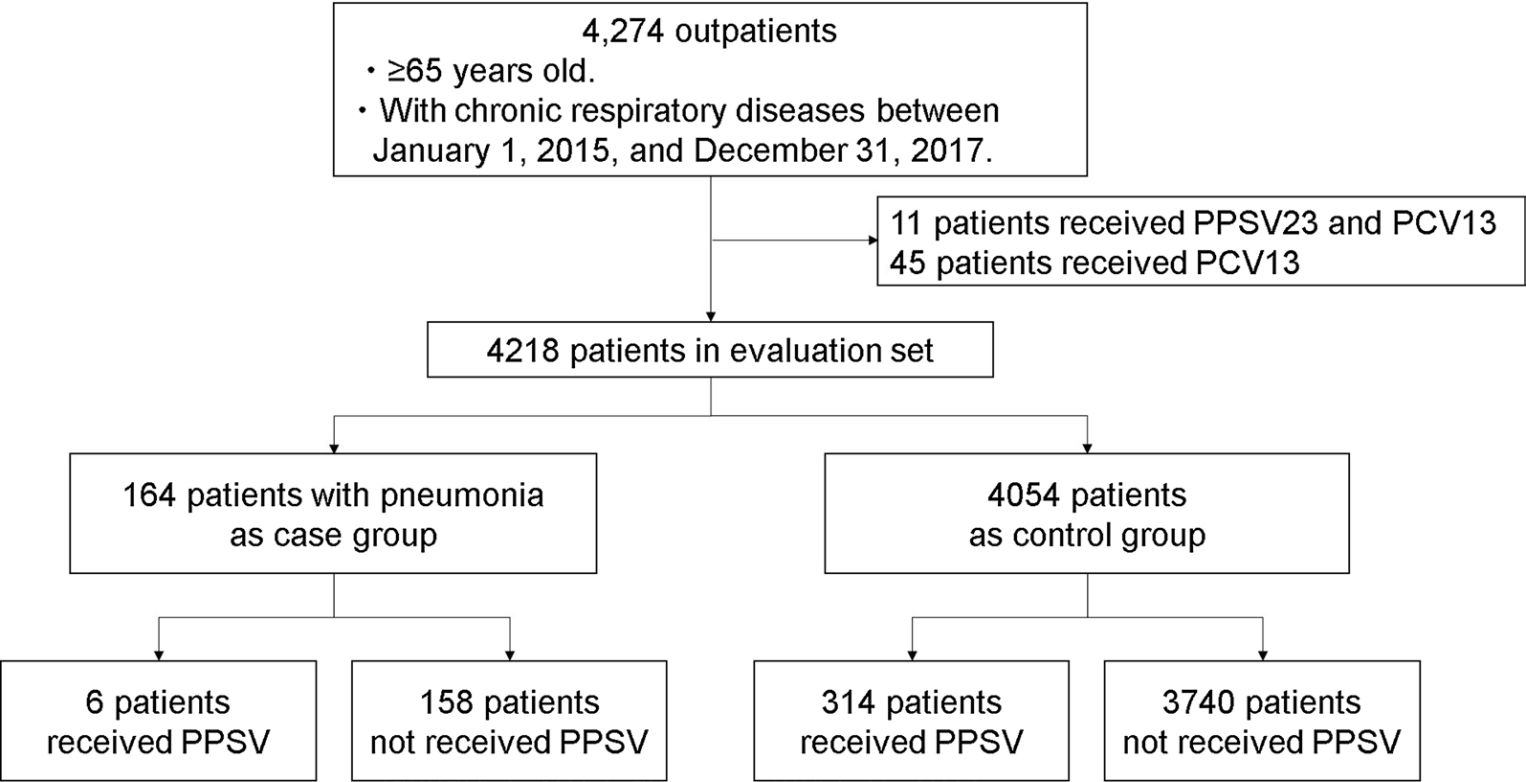
Patient flow. PPSV23: 23-valent pneumococcal polysaccharide vaccine, PCV13: 13-valent pneumococcal conjugate vaccine

Between January 1, 2015, and December 31, 2017, 4274 outpatients aged ≥ 65 years with chronic respiratory diseases visited our department. Of 4274, 45 had been vaccinated with PCV13 and 11 with PPSV23 and PCV13. These were excluded from the study, which included 4218 patients. The patient flow is shown in Fig. 1. A total of 320 patients received the PPSV23, while 3898 did not. Of the 320 vaccinated patients, 6 developed pneumococcal pneumonia, compared to 158 of the 3 898 unvaccinated patients.

The baseline characteristics of the case and control group on January 1, 2015, are shown in Table 1. Patients in the case group were more current-smokers, as well as had other chronic respiratory diseases, diabetes, chronic heart disease, and had higher use of systemic corticosteroids, than the control group. The number of patients in the case group with lung cancer was less than that of the control group.

### Effectiveness of the vaccine against pneumococcal pneumonia

**Fig. 2:**
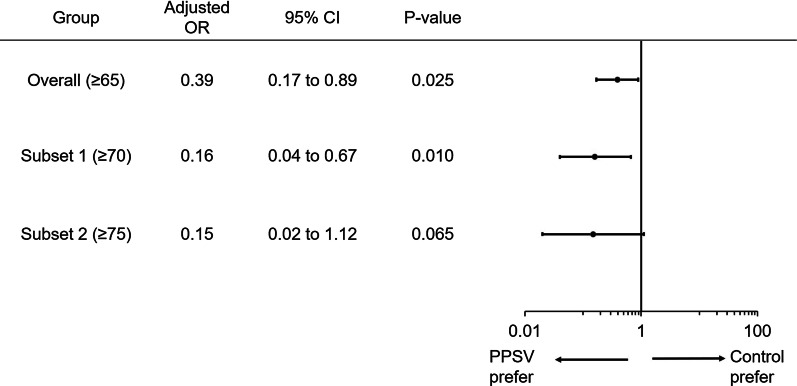
Effectiveness of vaccines in pneumococcal pneumonia in age-related subsets. OR, odds ratio; CI, confidence interval; PPSV23, 23-valent pneumococcal polysaccharide vaccine. The lower adjusted ORs in subsets of older patients were estimated, suggesting that the PPSV23 can be more effective for older patients. The adjusted OR was adjusted using the quantile category of propensity scores

Pneumococcal pneumonia prevention crude OR (95% CI) was 0.45 (0.20–1.03, *p* = 0.059), and the adjusted OR was 0.39 (0.17–0.89) (Fig. 2). There was a trend toward lower adjusted ORs in the subset restricted to only the more elderly: OR (95% CI): 0.39 (0.17–0.89) for those aged ≥ 65 years, 0.16 (0.04–0.67) for subset 1 (≥ 70 years), and 0.15 (0.02–1.12) for subset 2 (≥ 75 years). In the sensitivity analysis, the double-robust adjusted ORs in patients of ≥ 65, ≥ 70, and ≥ 75 years old were respectively 0.35, 0.14 and 0.13, thus, the above adjusted ORs were conservative and not overestimating.

### Age-specific effectiveness and patients’ characteristics

Age-specific patients’ characteristics were presented in Table 2. The crude ORs (95% CI) by age for vaccine efficacy against pneumococcal pneumonia in those aged 65 to < 70 years, 70 to < 75 years, and ≥ 75 years were 1.44 (0.49–4.22), 0.24 (0.03–1.74), and 0.17 (0.02–1.24), respectively (Table 2). The results of feeding the variables that were significant in Table 1 into a multivariate logistic regression model are shown in Additional file 1: Table 1. In this multivariate model, smoking, lung cancer, other chronic respiratory diseases, and systemic corticosteroid user were considered as risk factors. The ORs (95% CI) adjusted for these risk factors for each age group were 1.34 (0.45–4.02), 0.22 (0.03–1.59), and 0.17 (0.02–1.20) (Table 2).

**Table 2:** Age-specific effectiveness and patients’ characteristics

Variable and category (reference)	65 to < 70 years		70 to < 75 years		≥ 75 years		*p* value of interaction term with age category
	Case (n = 30)	Control (n = 974)	Case (n = 44)	Control (n = 1,085)	Case (n = 90)	Control (n = 1,995)	
PPSV23 (vs. absent)	4 (13.3)	94 (9.7)	1 (2.3)	97 (8.9)	1 (1.1)	123 (6.2)	0.090
Crude odds ratio (95% CI)	1.44 (0.49–4.22)		0.24 (0.03–1.74)		0.17 (0.02–1.24)		
Adjusted odds ratio^a^ (95% CI)	1.34 (0.45–4.02)		0.22 (0.03–1.59)		0.17 (0.02–1.20)		0.102
Male (vs. female)	19 (63.3)	585 (60.1)	33 (75.0)	696 (64.2)	61 (67.8)	1244 (62.4)	0.736
Smoking (vs. non-smokers)							0.727
Current smokers	21 (70.0)	522 (53.6)	33 (75.0)	578 (53.3)	54 (60.0)	968 (48.5)	
Ex-smokers	5 (16.7)	215 (22.1)	4 (9.1)	222 (20.5)	10 (11.1)	351 (17.6)	
Chronic respiratory diseases							
Asthma (vs. absent)	5 (16.7)	180 (18.5)	11 (25.0)	189 (17.4)	14 (15.6)	316 (15.8)	0.506
COPD (vs. absent)	6 (20.0)	210 (21.6)	16 (36.4)	282 (26.0)	20 (22.2)	467 (23.4)	0.360
Lung cancer (vs. absent)	9 (30.0)	415 (42.6)	19 (43.2)	409 (37.7)	17 (18.9)	770 (38.6)	**0.013**
Interstitial pneumonia (vs. absent)	7 (23.3)	167 (17.2)	8 (18.2)	195 (18.0)	14 (15.6)	340 (17.0)	0.646
NTM (vs. absent)	11 (36.7)	167 (17.2)	10 (22.7)	191 (17.6)	27 (30.0)	388 (19.5)	0.405
Others chronic respiratory diseases^b^ (vs. absent)	5 (16.7)	87 (8.9)	4 (9.1)	118 (10.9)	28 (31.1)	332 (16.6)	0.218
Diabetes (vs. absent)	9 (30.0)	253 (26.0)	26 (59.1)	350 (32.3)	30 (33.3)	606 (30.4)	**0.036**
Chronic heart disease (vs. absent)	12 (40.0)	350 (35.9)	25 (56.8)	504 (46.5)	58 (64.4)	1036 (51.9)	0.736
Chronic kidney disease (vs. absent)	0	28 (2.9)	3 (6.8)	25 (2.3)	0	64 (3.2)	0.998
Systemic corticosteroid user (vs. absent)	15 (50.0)	311 (31.9)	26 (59.1)	363 (33.5)	24 (26.7)	515 (25.8)	**0.029**

Bold value indicates statistical significance

^a^The odds ratios for PPSV23 were calculated for adjusting smoking, lung cancer, other chronic respiratory diseases, and systemic corticosteroid user

^b^Other chronic respiratory diseases included chronic pulmonary aspergillosis, old pulmonary tuberculosis, sarcoidosis, and chronic cough

CI, confidence interval; COPD, chronic obstructive pulmonary disease; NE, not evaluated; NTM, non-tuberculous mycobacteriosis; PPSV23, 23-valent pneumococcal polysaccharide vaccine

The variables containing categories with different rates of pneumococcal pneumonia according to age according to the interaction term test were lung cancer (*p* = 0.013), diabetes mellitus (*p* = 0.036), and presence of systemic corticosteroid use (*p* = 0.029) (Table 2).

## Discussion

This study found that the PPSV23 prevented pneumococcal pneumonia in older patients (age ≥ 65 years) with chronic respiratory diseases, and could be more effective for the elderly (patients aged ≥ 70 years). To the best of our knowledge, this is the first real-world study that assesses the effectiveness of the PPSV23 in older patients with chronic respiratory disease in a single center.

In previous studies, the effectiveness of the vaccine against pneumococcal pneumonia was controversial. In the results of a meta-analysis of 18 randomized trials [5], the PPSV23 reduced the risk of IPDs such as bacteremia, meningitis, and bacteremic pneumonia (OR [95% CI]: 0.26 [0.15–0.46]) and pneumococcal pneumonia (0.46 [0.25–0.84]). However, some studies reported that the PPSV23 did not reduce pneumococcal pneumonia [6, 7, 10]. The EVAN-65 study in community-dwelling patients [11] showed that the hazard ratios (HR) for the risk of pneumococcal pneumonia in vaccinated patients compared with non-vaccinated patients were not different (HR [95% CI]: 0.61 [0.35–1.06]). Similarly, another study in elderly patients with chronic respiratory diseases showed no difference (0.76 [0.30–1.90]) [12]. These conflicting results can be due to the difficulties of accurately diagnosing pneumococcal pneumonia and the use of non-validated diagnostic tests [13]. In this study, we included non-invasive cases according to specific diagnostic criteria of pneumonia, urine pneumococcal antigen, and sputum culture, and we are convinced that these results are close to correct.

One previous case–control study suggested that 85–90% of adults aged 55 and younger achieved the prevention of invasive pneumococcal infections, but this effect was reduced with increasing age, and no protection was shown in the population aged ≥ 80 years [14]. Another study suggested that the prevention of community-onset pneumococcal pneumonia was effective in people aged 65-75 years but not effective in those aged ≥ 75 years in Japan [15]. The population-based retrospective cohort study in Germany reported that the prevention of pneumococcal pneumonia was not effective at all in people aged ≥ 80 years [16]. These studies suggest that poorer effectiveness might be due to immunosenescence, which refers to the decline of the immune system associated with aging [17]. However, this study found that the OR was much decreased in older people, which could imply that older people can acquire antibodies with the PPSV23 vaccination, and we proposed that older people be re-vaccinated because of an anticipated decline in the effectiveness of the vaccine over time.

Almost all patients with chronic respiratory diseases in this study had risk factors for pneumonia [18]. Previously, most studies on the PPSV23 targeted nursing home residences or healthy adults, and controversial results have been reported on non-invasive pneumonia [8]. Our findings suggest the importance of vaccinating chronic respiratory patients in clinical practice.

There were several limitations. First, this study was a retrospective single-center study, and a complete confounding adjustment was not done. Second, we did not assess the severity of the underlying diseases. The study population was biased, consisting mainly of moderate to severe disease cases. Third, we did not regularly identify the serotype of each pneumococcal pneumonia. Fourth, the vaccination status of unvaccinated persons was defined based on the lack of medical records of vaccination, and some vaccinated patients may have been misclassified as unvaccinated. Fifth, in this study, we were not able to investigate the patients' history of seasonal influenza vaccination. This may affect the risk of pneumococcal infections.

In summary, the PPSV23 can be useful in preventing pneumococcal pneumonia among the elderly with chronic respiratory diseases.

## Supplementary Information

**Additional file 1: Table 1.** The result of multivariable logistic regression analysis to identify risk factors for pneumococcal pneumonia.

## Data Availability

The datasets used and/or analysed during the current study are available from the corresponding author on reasonable request.
