# Supplementary material for: Effectiveness of a 23-valent pneumococcal polysaccharide vaccine for the prevention of pneumococcal pneumonia in the elderly with chronic respiratory diseases: a case–control study of a single center
Source: BMC Pulm Med. 2021 Apr 16;21:123. doi: 10.1186/s12890-021-01491-w (PMC8051051; doi:10.1186/s12890-021-01491-w)
Supplement: Supplementary file 1 — Additional file 1: Table 1. The result of multivariable logistic regression analysis to identify risk factors for pneumococcal pneumonia. [file 12890_2021_1491_MOESM1_ESM.docx]

**Supplementary File**

**Supplementary Table 1.** The result of multivariable logistic regression analysis to identify risk factors for pneumococcal pneumonia.

| Variable (reference) | Category | Multivariable regression model | | |
| --- | --- | --- | --- | --- |
|  |  | OR | 95% CI | P-value |
| Smoking (vs. non-smokers) | Current smokers | 0.93 | 0.52-1.66 | **0.002** |
|  | Ex-smokers | 1.81 | 1.23-2.66 |  |
| Lung cancer (vs. absent) | Presence | 0.57 | 0.40-0.83 | **0.004** |
| Others chronic respiratory diseases^†^ (vs. absent) | Presence | 1.74 | 1.17-2.60 | **0.007** |
| Diabetes (vs. absent) | Presence | 1.21 | 0.87-1.69 | 0.264 |
| Chronic heart disease (vs. absent) | Presence | 1.29 | 0.92-1.79 | 0.136 |
| Systemic corticosteroid user (vs. absent) | Presence | 1.48 | 1.06-2.08 | **0.022** |

^†^Other chronic respiratory diseases included chronic pulmonary aspergillosis, old pulmonary tuberculosis, sarcoidosis, and chronic cough.

CI: confidence interval, OR, odds ratio.
